# Defining the effects of age and gender on clinical immune response to cancer vaccination

**DOI:** 10.1186/2051-1426-2-S3-P59

**Published:** 2014-11-06

**Authors:** Adriana Ramirez, Nolan Wages, Mark E Smolkin, Craig L Slingluff

**Affiliations:** 1 University of Virginia, Charlottesville, United States

## Objectives

Cancer vaccines have promise as monotherapy or as part of combination immunotherapy regimens. Age and gender implications on immune response to cancer vaccinations have not previously been well described. There is uncertainty about including elderly patients as study participants in vaccine trials because of the decline in immune response with increasing age [1,2]. Compelling evidence has also demonstrated gender differences in immune response associated with injury and vaccination for infectious diseases [3,4]. We hypothesized younger age and female gender may be predictive of higher rates of immune response to a multipeptide cancer vaccine. Data collected from three clinical trials evaluating T cell response to a polyvalent melanoma peptide vaccine provide an opportunity to assess the association of immune response with age and gender.

## Methods

Patients with resected stage IIB-IV melanoma were enrolled in three clinical trials: Mel43, Mel44, and Mel48, in which they received 6 vaccinations with 12 Class I MHC restricted peptides from melanocytic differentiation antigens and cancer testis antigens with each of various adjuvants. T cell responses were detected by direct IFN-gamma ELIspot assay. Clinical data, including age and gender, were collected. The cumulative incidence in immune response over time was plotted for each variable. Chi-squared analysis was performed on the number of immune responders by age and gender.

## Results

A total of 327 patients were evaluated for immune response measurable by Week 7. Immune responses were detected in 111/224 (49.5%) males and 49/103 (47.6%) females, p = 0.74. Immune responses were detected in 130/249 (53%) in patients less than 64 years old, 30/78 (38.5%) older patients (p = 0.026 by cumulative incidence over time; p = 0.034 by Chi-squared analysis). Age greater than 64 accounted for a 14.5 % decrease in immune response.

## Conclusion

While our study shows a measurable decline in immune response with increasing age, a significant percentage of elderly patients do develop immune responses to vaccination. Meanwhile, gender was not shown to affect immune response despite evidence suggesting an immunoprotective association with female gender [3].This information should be considered in patient selection for cancer vaccines. In particular, elderly patients should be made aware of this difference prior to undergoing vaccination but should not be excluded on the basis of age.
